# High impact resistance in graphyne†

**DOI:** 10.1039/c9ra09685j

**Published:** 2020-01-09

**Authors:** Yang Yang, Qiang Cao, Yang Gao, Shuting Lei, Sheng Liu, Qing Peng

**Affiliations:** The Institute of Technological Sciences, Wuhan University Wuhan 430072 China caoqiang@whu.edu.cn; Key Laboratory of Hydraulic Machinery Transient, Ministry of Education, Wuhan University China; Science and Technology on Special System Simulation Laboratory, Beijing Simulation Center Beijing 100854 PR China; Physics Department, King Fahd University of Petroleum & Minerals Dhahran 31261 Saudi Arabia qpeng.org@gmail.com http://qpeng.org

## Abstract

Graphyne was recently facilely synthesized with superior mechanical and electrical performance. We investigate the ballistic protection properties of α-, β-, δ-, and γ-graphyne sheets using molecular dynamics simulations in conjunction with elastic theory. The velocities of the in-plane elastic wave and out-of-plane cone wave are obtained by both membrane theory and molecular dynamics simulations. The specific penetration energies are approximately 83% that of graphene, indicating high impact resistance. γ-Graphyne has high sound wave speeds comparable to those of graphene, and its Young's modulus is approximately 60% that of graphene. δ-Graphyne has the highest cone wave speed among the four structures, while α-graphyne possesses the highest penetration energy and impact resistance at most tested projectile speeds. Our results indicate that graphyne is a good protective structural material.

## Abstract 2

Graphyne was recently facilely synthesized with superior mechanical and electrical performance.

## Graphical abstract

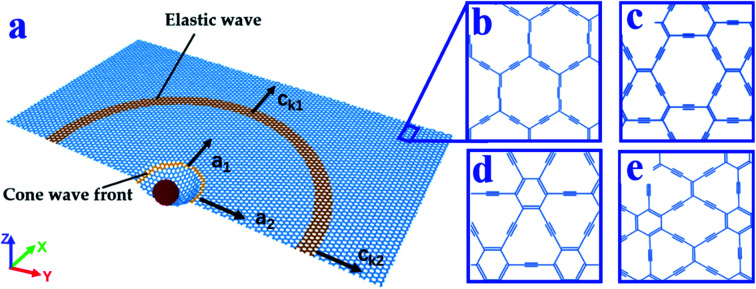

## Introduction

Armor protection materials have attracted extensive attention throughout human history. At present, the strengths of ideal lightweight materials used in ballistic protection materials such as Kevlar, Spectra, and Dyneema are only several GPa.^1–3^ To overcome the limitations of these traditional protection materials, new materials like graphene, carbon nanotubes, and polymers have recently been studied as protective materials.^4–6^ Efforts have been made to identify other ballistic protection materials,^7–9^ among which graphene has become a focal point^4,10,11^ due to its super-high strength.^12^ Meanwhile, additional carbon allotropes have attracted attention due to the potentially excellent material properties.^13^

**Fig. 1:**
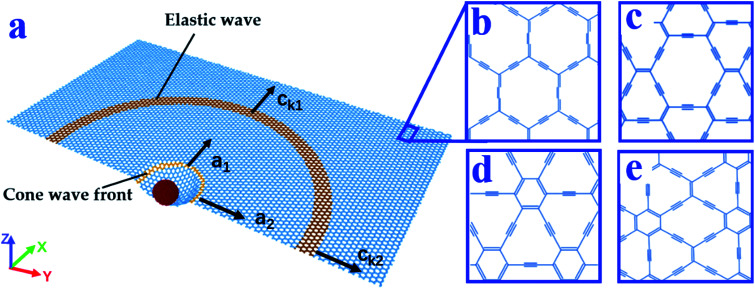
(a) Schematic diagram of the impact. The yellow and orange rings represent the positions where the cone wave front and elastic wave propagate to, respectively. Structures of (b) α-graphyne, (c) β-graphyne, (d) δ-graphyne, and (e) γ-graphyne.

As a hybrid sp and sp^2^ carbon allotrope, graphyne was first proposed by Baughman *et al.* as a theoretically possible structure.^14^ By replacing the sp^2^ carbon bonds in graphene with acetylenic linkages in proportions of 100%, 66.67%, 41.67%, and 33.33%, four kinds of graphyne (α-, β-, δ-, and γ-graphyne, respectively) are obtained.^15,16^ The atomic structures of these graphynes are shown in Fig. 1(b)–(e). In previous studies, graphyne showed attractive electronic and optical properties.^17–20^ Very recently, the mechanical properties of graphynes with defects were studied, indicating that graphynes with minor defects still retain most of the strengths of graphyne.^21^ In addition to extensive theoretical investigations, graphyne has been successfully synthesized^22^ using numerous synthetic methods, including surface-assisted covalent synthesis,^23^ Glaser–Hay coupling reaction,^24^ and morphology-controlled synthesis.^25^ Additionally, a solution-phase van der Waals epitaxial strategy has been proposed to synthesize δ-graphyne in a facile manner.^26^ This approach may be helpful for practical applications of graphyne.

Impact and penetration tests are designed to characterize the security mechanisms and effectiveness of a material against attacks.^27–29^ Penetration tests have shown that multilayer graphene has potential in ballistic energy dissipation.^4^ Impact and penetration tests have also been applied to study carbon allotropes.^30–33^

However, studies on graphyne's ballistic impact resistance in protective applications are rare.^34^ This study aims to bridge this gap. To capture the strain rate characteristics of graphyne, molecular dynamics (MD) simulations were conducted. We studied the ballistic protection properties of four types of graphyne monolayers at room temperature. The propagation speeds of both the elastic and cone waves produced during ballistic impact tests can reflect the energy delocalization and momentum transfer ability. We obtained the Poisson's ratios and Young's moduli of the graphynes *via* tensile tests. According to membrane theory, we derived the theoretical speeds of the elastic and cone waves. MD simulations of graphyne monolayers under supersonic impacts were then conducted to acquire the wave speeds. To develop a more intuitive understanding, we studied the specific penetration energies (SPEs) and analyzed the fracture patterns of graphynes.

## Materials and methods

MD simulation is a well-established method for atomistic modeling and is especially suitable for high-shock-wave simulations and impact simulations.^35^ In this study, MD simulation was conducted using the Large-scale Atomic/Molecular Massively Parallel Simulator (LAMMPS) software package.^36^ The interaction between the diamond ball and graphyne monolayer was described by a Lennard-Jones potential.^37^ For the interactions between the carbon atoms inside monolayer graphyne and the diamond ball, the adaptive intermolecular reactive bond order (AIREBO) potential was adopted.^38^ To demonstrate the validity of our simulations, we first performed a MD simulation of graphene and compared the results with those of Haque *et al.*^10^ We imitated Haque's method of calculating the elastic wave speed, cone wave speed, and penetration energy. The difference between this study and Haque *et al.* lies in the dimensions of our target materials (ours are a little smaller to save computational resources), the projectile ball's material, and the environmental temperature. We set the velocity of the diamond ball to 3.5 km s^−1^, the same as in Haque's study. The cone wave propagation speeds were 4.04 and 3.71 km s^−1^ along the *X* and *Y* directions, respectively, which agrees well with those in Haque's study (3.940 and 3.917 km s^−1^, respectively). Thus, it is reasonable to compare our data for graphyne directly with Haque's graphene data.

### Tensile tests of graphyne sheets

n the tensile tests, the dimensions of the monolayers of α-, β-, δ-, and γ-graphyne were 12 × 10, 11 × 11, 11 × 11, and 11 × 12 nm^2^, respectively. The thickness of each monolayer was 0.334 nm.^39^ The simulation temperature was maintained at 300 K using a Nose–Hoover thermostat.^40,41^ The time step was 0.0005 ps. The structures were fully relaxed using the isothermal–isobaric ensemble (NPT) ensemble. Uniaxial tensile loadings were then applied along the *X* (or *Y*) direction. The strain rate was set to 0.5 × 10^9^ s^−1^.

From the tensile tests, we obtained the Young's moduli (*E*_1_, *E*_2_) as *σ*_1_ = *E*_1_*ε*_1_, *σ*_2_ = *E*_2_*ε*_2_ and Poisson's ratios (*ν*_12_, *ν*_21_) 
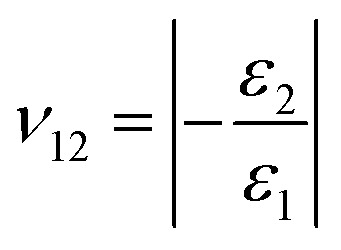
, 
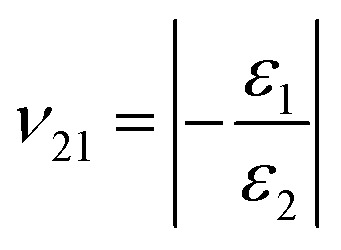
 of the four types of graphyne, where, *E*_1_ and *E*_2_ are the Young's moduli in the *X* and *Y* directions respectively; *σ*_1_ and *σ*_2_ are the stresses in the *X* and *Y* directions, respectively; *ε*_1_ and *ε*_2_ are the strains in the *X* and *Y* directions, respectively; and *ν*_12_ and *ν*_21_ are the Poisson's ratios along the *X* and *Y* directions, respectively.

### Supersonic ballistic impact tests on graphyne sheets

In the ballistic impact tests, the dimensions of the α-, β-, δ-, and γ-graphyne monolayers were 62 × 64, 70 × 70, 67 × 60, and 69 × 67 nm^2^, respectively. These four atomic systems are called initial systems (distinguished from comparison systems in the following penetration tests). The diamond projectile was set as a rigid ball to avoid the effect of its deformation. The diameter of the ball was 3.57 nm. The original position of the ball was set at 8.285 nm above the center of the graphyne flake. The starting speed of the diamond projectile was set in a range of 0.5–2.5 km s^−1^. To fix the position of the graphyne film in the *Z* direction, the outermost ring of atoms of the film were clamped. The graphyne flakes were thermodynamically equilibrated at 300 K using the NVT (canonical) ensemble for 20 ps. The NVE (micro-canonical) ensemble was then applied for the impact process.

To study in-plane elastic wave (sound wave) and out-of-plane cone wave propagation, several points along the *X* and *Y* directions were marked for displacement observation, as shown in Fig. S1 in the ESI.† Starting at 2 nm from the center of the graphyne monolayer, points were marked at intervals of 2 nm along the *X*-direction from *X*1 to *X*9. At 20 nm from the center, points were marked every 3 nm along the *X* direction (*X*10 to *X*13). Points in the *Y* direction were arranged the same way as points in the *X* direction. Points 1–9 in both directions were used to calculate the velocity of cone wave propagation, while points 10–13 were used to evaluate elastic wave propagation. The wave velocity was calculated by dividing the distance between two points by the average time that the wave took to travel between two adjacent points. The displacement–time image derived from the simulation results provided the time information needed in the velocity calculation.

According to membrane theory,^42–45^ the theoretical elastic wave speeds (*a*_1_, *a*_2_) can be calculated as 
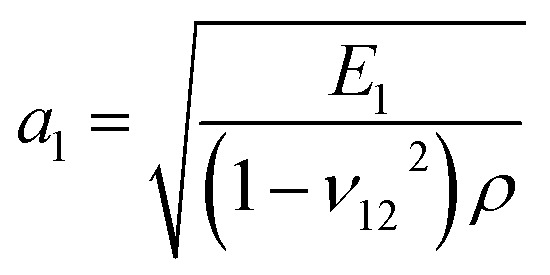
 and 
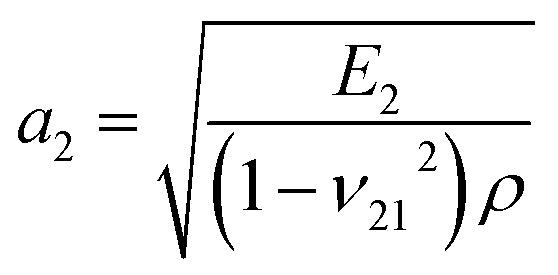
, where *ρ* is the mass density of graphyne.

According to the derivation of Phoenix & Porwal,^42^ the cone wave speeds (*c*_k1_, *c*_k2_) can be calculated as 
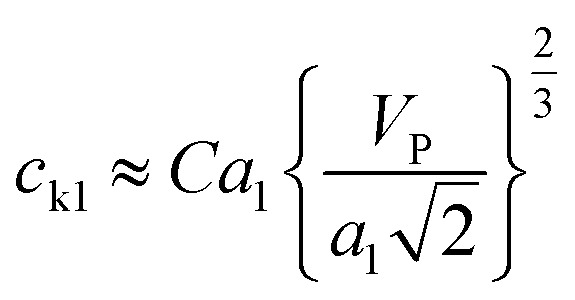
 and 
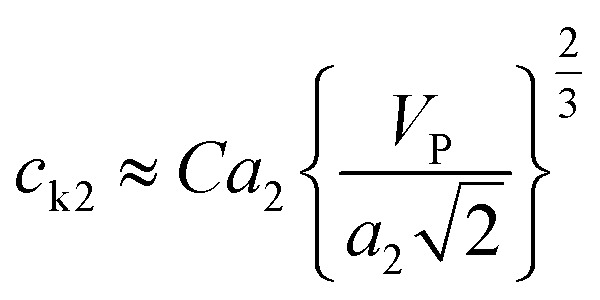
, where *C* is a constant that depends on the structure of graphyne and was first set to 1, and *V*_P_ is the impact velocity of the diamond projectile.

To more intuitively describe the energy absorption properties of graphyne, we also calculated the penetration energy. We first used a new atomic system called the comparison system (distinguished from the initial system) for convenience of comparison with graphene.^11^ It is worth mentioning that the dimensions of the comparison system were much different than those of the initial system. In comparison system, both specific penetration energy values for graphyne and graphene are simulated by us, and compared with each other. We used the same ball radius of 35.7 Å. All graphyne monolayers were sized to approximately 40 × 40 nm. The speed of the diamond ball was set at 5 km s^−1^. For the initial system, we studied the penetration energy with ball speeds of 2.5, 3, 4, and 5 km s^−1^. According to the conservation of energy, the energy transferred to the graphyne sheet (*E*^GS^_T_) can be calculated as *E*^GS^_T_ = *E*^I^_P_ − *E*^R^_P_, where *E*^I^_P_ and *E*^R^_P_ are the kinetic energies of the projectile before and after the impact, respectively. SPE can be calculated as SPE = *E*^GS^_T_/*m*, where *m* is the graphyne mass involved in the projectile penetration and can be calculated as *m* = π*R*^2^*ρ*, where *R* is radius of the ball, and *ρ* is the corresponding graphyne area density.

## Results

### Young's modulus and Poisson's ratio

The Young's moduli and Poisson's ratios of α-, β-, δ-, and γ-graphynes are summarized in Table 1. Our results agree well with previous studies.^17,46^ Among the graphynes, δ- and γ-graphyne had the highest strengths (476.9 and 538.5 GPa in the *X* direction, respectively, about half that of graphene). The densities of α-, β-, δ, and γ-graphynes were 1135.57, 1380.27, 1742.46, and 1649.81 kg m^−3^, respectively, much smaller than that of graphene^4^ (2200 kg m^−1^; Table 1). Meanwhile, the Young's modulus was closely correlated with mass density. Considering the density and strength, γ-graphyne was the strongest among the four graphyne structures.

**Table 1:** Tensile elastic properties of monolayer graphynes. The values for graphene are from Lee *et al*.^4^ and Haque *et al*.^10^

Graphyne type	Density (kg m^−3^)	Young's modulus (GPa)		Poisson's ratio	
		*E*_1_	*E*_2_	*ν*_12_	*ν*_21_
α-Graphyne	1135.6	178.5	158.3	0.59	0.65
β-Graphyne	1380.3	339.7	321.9	0.37	0.41
δ-Graphyne	1742.5	476.9	379.7	0.25	0.32
γ-Graphyne	1649.8	538.5	518.2	0.44	0.33
Graphene	2200 (ref. 4)	883 (ref. 10)	893 (ref. 10)	0.33 (ref. 10)	0.31 (ref. 10)

### Elastic wave speed

**Fig. 2:**
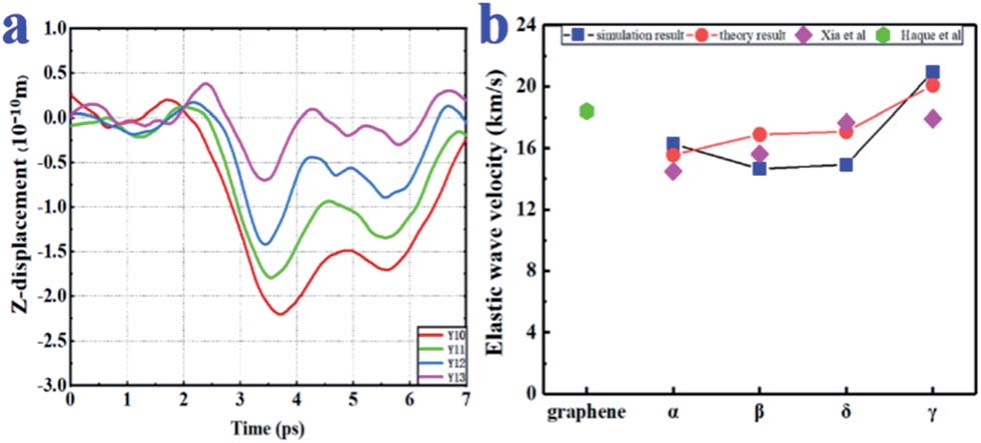
(a) Displacement–time plots in the *Y*-direction (points *Y*10–*Y*13) for α-graphyne under a 2.0- km s^−1^ impact. (b) Comparison of the theoretical and simulated values of elastic wave propagation velocity in the *X* direction. The simulated values of both Haque *et al.*^10^ and Xia *et al.*^34^ are shown for comparison.

The elastic wave (or sound wave) represents the propagation process of the vibration generated during the impact. The speed of the elastic wave reflects the ability of the material to transmit momentum. As shown in Fig. 2b, the speeds of elastic wave propagation in the four graphynes were similar. Among the four graphynes, γ-graphyne had the largest theoretical speeds in both the *X* and *Y* directions (*a*_1_ = 20.08 km s^−1^; *a*_2_ = 18.80 km s^−1^). More data are presented in Table S1† and shown in Fig. S2 in the ESI.† It is worth noting that our theoretical elastic wave speeds are still lower than those of graphene in the literature (*a*_1_ = 21.22 km s^−1^; *a*_2_ = 21.19 km s^−1^).^10^

The displacements of points *X*10–*X*13 points in the *Z* direction are shown as a function of time for α-graphyne in Fig. 2a. The simulated elastic wave speeds *a*_1_ and *a*_2_ were calculated by dividing the inter-point distance by the difference in arrival time. The trends of the simulated wave speeds in both the *X* and *Y* directions were consistent with the theoretical values (Fig. 2b), demonstrating the validity of our simulations. The simulation values were smaller than the theoretical values, which may be due to the anisotropic nature of graphyne.^10^ For instance, the simulated elastic wave speeds of δ-graphyne in the *X* and *Y* directions were 14.93 and 12.41 km s^−1^, respectively, which agree well with Peng's first-principles study^17^ but are smaller than those determined based on membrane theory (17.08 and 15.58 km s^−1^, respectively).

It is worth mentioning that the elastic wave propagation speeds of graphynes are much higher than those of traditional engineering materials (*e.g.*, 5.2 km s^−1^ for steel and 5.5–7 km s^−1^ for rock), indicating the outstanding impact resistance of graphyne.

### Cone wave speed

**Fig. 3:**
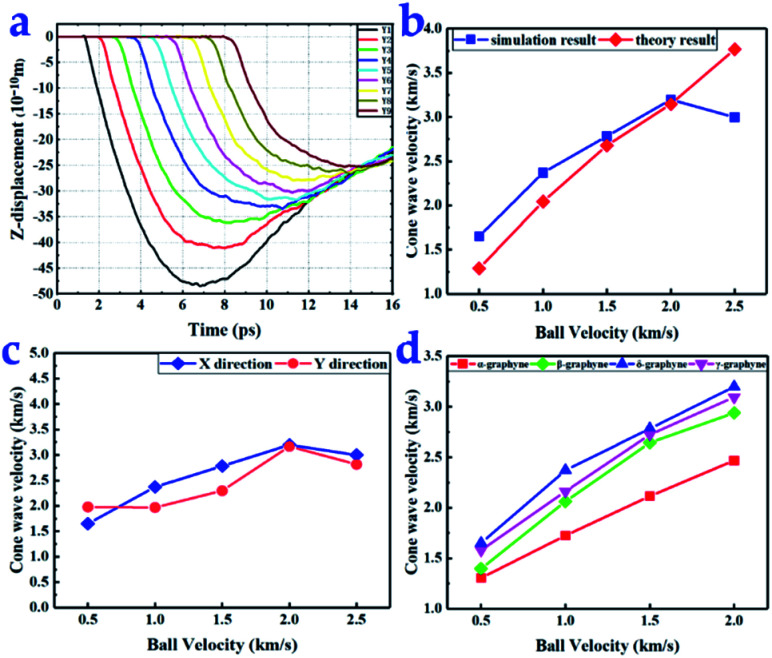
(a) Displacement–time diagram in the *Z*-direction (points *Y*10–*Y*13) for α-graphyne under a 2.0 km s^−1^ diamond projectile impact. (b) Comparison of the theoretical and simulated values of δ-graphyne cone wave velocity in the *X* direction. (c) δ-Graphyne cone wave propagation velocities in the *X* and *Y* directions. (d) Cone wave propagation velocity in the *X* direction as a function of ball velocity.

Cone waves caused far larger displacement (*Z* direction) and stronger disturbance than elastic waves (*X* and *Y* directions), as observed in the ESI videos.† This is because the ball impacted graphyne along the *Z* direction, resulting in considerable momentum transfer. The cone wave propagation speed plays an important role in impact energy dissipation.^47^ Using the displacement–time diagram for nine points (Fig. 3a), we calculated the cone wave propagation speeds of the graphynes.

When the speed of the diamond projectile did not exceed 2.0 km s^−1^, the propagation speed of the graphyne cone waves increased with increasing projectile velocity (Fig. 3b). However, the cone wave propagation speed dramatically decreased when the ball velocity reached 2.5 km s^−1^, the velocity at which all four types of graphyne monolayers were broken. When a ball impacts graphyne, it will either penetrate the flake or be bounced back. Based on the law of conservation of momentum, a smaller proportion of momentum is transferred to graphyne in the case of penetration than in the case of a bounce back, resulting in a dramatic decrease in cone wave speed. More detailed data are shown in Table S2 of ESI.†

The cone wave propagation speeds were different in the *X* and *Y* directions, as shown in Fig. 3c, illustrating anisotropic behavior. The cone wave propagation speeds of the tour types of graphyne in the *X* direction are compared in Fig. 3d. Among the graphynes, δ-graphyne possessed the largest cone wave propagation speed. When the velocity of the ball reached 2 km s^−1^, the speed of the cone wave reached the maximum of 3.2 km s^−1^. Meanwhile, α-graphyne had the lowest cone wave propagation speed among the graphyne structures, and the difference became more obvious as the impact velocity increased.

### Penetration energy

**Fig. 4:**
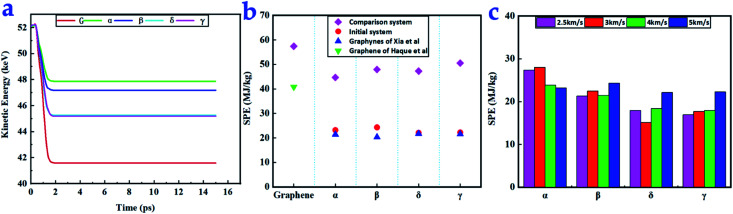
(a) Change in kinetic energy over time for a projectile impacting graphynes and graphene (red line) at an impact velocity of 5 km s^−1^ (b) SPE values of graphynes and graphene for a 5 km s^−1^ impact velocity along with corresponding values from Haque *et al.*^10^ and Xia *et al.*^34^ (c) SPE values of the initial systems for different impact velocities from 2.5 to 5 km s^−1^.

The penetration energy is the energy absorbed from a projectile during penetration and reflects the impact resistance of the material. The kinetic energies of the ball over time in the penetration tests with the four graphynes compared with the data for graphene in Fig. 4a. The sharp decreases in kinetic energy reflect the absorption of impact energy by the materials. Graphene absorbs most of the kinetic energy upon impact. Among the four graphynes, δ- and γ-graphyne showed excellent performance in absorbing kinetic energy; they absorbed almost 65% that of graphene. In contrast, α- and β-graphynes absorbed 40% and 46% of the kinetic energy of graphene, respectively.

We adopted SPE for convenience and to allow comparison with various materials using a fixed projectile. The SPEs of the five tested materials were similar. The SPEs of the comparison system (ball radius = 3.57 nm) and initial system (ball radius = 1.785 nm) are presented in Fig. 4b. When the ball radius was 3.57 nm, graphene had the largest SPE (57.4 MJ kg^−1^) among all tested materials. This value is higher than that reported by Haque *et al.* (40.8 MJ kg^−1^) because the radius of our ball was 1.33 times that used by Haque.^10^ The four types of graphyne had similar SPEs with an average of 47.6 MJ kg^−1^, which is 83% that of graphene. This indicates that the different carbon atom arrangement in graphyne did not have a significant influence on the SPE of the film when the projectile (*R* = 3.57 nm) speed was 5 km s^−1^. However, the ball radius can significantly influence the SPE.

For the initial system, β-graphyne had the largest SPE (24.4 MJ kg^−1^) among the four graphynes under the 5 km s^−1^ impact velocity. However, at all other ball speeds, α-graphyne had the largest SPE. Graphene in the initial system was not penetrated, again indicating that graphene is much stronger than graphyne; thus, there are no corresponding points in those figures. Xia *et al.* found that the graphynes had different performances, with δ-graphyne performing best.^34^

The SPEs of our initial system under impact velocities of 2.5, 3, 4, and 5 km s^−1^ impact are shown in Fig. 4c. As the impact velocity increased, the SPE first decreased and then increased for all four graphyne types. This phenomenon is in accordance with the prediction of Lee *et al.*,^4^ who found that when the ball radius is much larger than the thickness of the film, the penetration energy can be expressed as 
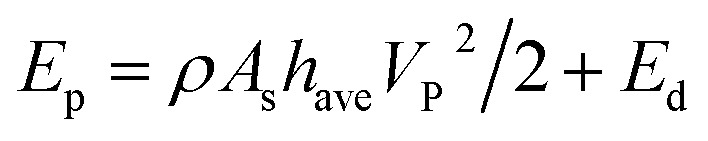
, where the first term represents the minimum inelastic energy transferred to the target with *A*_s_ = π*R*^2^; and the second term reflects the impact energy delocalization ability of the material. Thus, SPE can be expressed by 
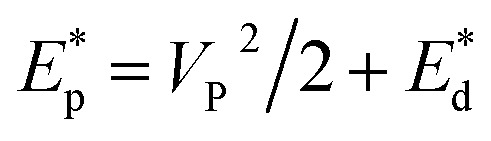
. The physical quantities with asterisks are divided by the mass of the involved graphyne atoms to facilitate comparison. We found that a higher material density corresponded to a better SPE. The SPEs of both δ- and γ-graphyne increased almost continuously with increasing impact velocity.

It is worth noting that all our simulated values of graphene SPE were an order of magnitude higher than the reported experimental values.^4^ This phenomenon can be explained using a scaling law.^11,48–50^ According to Bizao *et al.*, as the number of layers increases, the difference between the simulated and experimental results decreases.^11^

### Impact damage mode

**Fig. 5:**
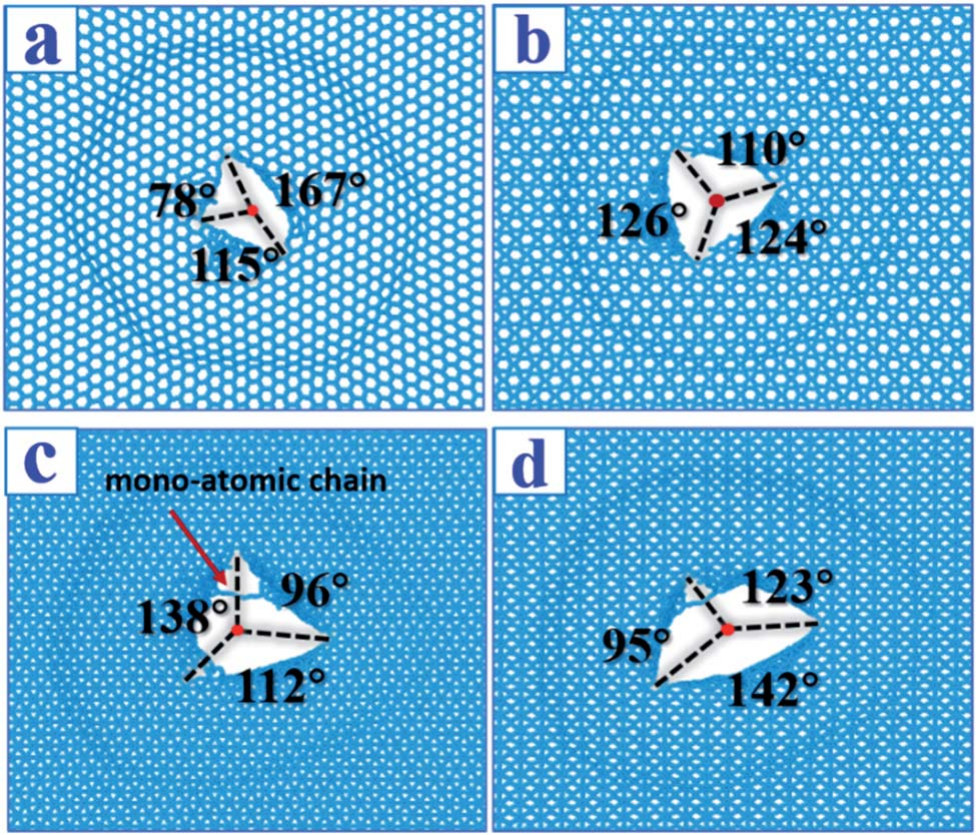
Comparisons between our room temperature fracture patterns and low temperature patterns (Xia) after impact for graphynes. Impact velocity is 2.5 km s^−1^. The left line is our patterns, the right is from Xia *et al.*^34^ (a) α-Graphyne; (b) β-graphyne; (c) δ-graphyne; (d) γ-graphyne. For whole penetration process, please see ESI videos.†

After the penetration tests, the fracture patterns were analyzed (Fig. 5). The fracture geometry of α-graphyne resembled a boat, and the radius of the externally tangent circle (excircle) was 2.78 Å, the smallest among the graphynes. The boat can be divided into three parts with apex polygon angles of 78°, 115°, and 167°. This boat-shape fracture pattern generates a loss of 55 atoms of graphyne, the largest loss among the graphyne monolayers. The fracture geometries of the β-, δ-, and γ-graphynes resembled equilateral triangles with different excircle radii of 3.53, 4.29, and 4.18 Å, respectively. The atom losses corresponding to the fractures of β-, δ-, and γ-graphyne were 1, 12, and 0, respectively. The detailed apex polygon angles of β-, δ-, and γ-graphyne are shown in Fig. 5. Although δ- and γ-graphyne had the largest excircle radii, they also contained mono-atomic chains in their fracture patterns. These chains prevented the fracture zones from expanding further, as observed in our ESI videos.† In this study, δ- and γ-graphynes contained two and one mono-atomic chains, respectively. In contrast, in Xia's study, δ- and γ-graphyne had one and zero chains, respectively,^34^ suggesting that a relatively high temperature may facilitate the formation of mono-atomic chains.

In the penetration tests at room temperature, the fracture patterns obtained in this study are slightly different from those reported by Xia *et al.* at 10 K, although they are generally similar.^34^ This suggests that fracture patterns are temperature insensitive because the penetration process is super-fast and athermal in nature.

## Conclusions

We investigated the ballistic protection properties of four types of graphyne using MD simulation in conjunction with elastic theory. Tensile tests showed that the strengths of δ- and γ-graphyne were about half that of graphene, while the densities of graphyne were also lower than that of graphene. The sound wave speeds of graphyne were relatively high, reaching 17.08 and 15.58 km s^−1^ for γ-graphyne in the *X* and *Y* directions, respectively. The speed of cone wave propagation increased as the projectile velocity increased. When the velocity of the diamond projectile reached 2.5 km s^−1^, the graphyne sheets were penetrated, and the cone wave speed decreased significantly. Among the four types of graphyne, the cone wave propagation speed was highest for δ-graphyne at various projectile speeds. Penetration tests indicated that the SPEs of all graphynes were 17% lower than that of graphene. Although the ballistic protection properties of α-graphyne were worse than those of the other three graphyne types, α-graphyne had the largest SPE in the tested projectile speed range along with minimal fracturing. The results indicate that graphyne monolayers transfer energy faster than conventional materials such as steel. Although graphyne is still inferior to graphene in many aspects, the properties of graphyne are of the same magnitude as those of graphene. Graphyne is lightweight, can be easily synthesized, and exhibits fast stress-wave propagation, giving it great potential for constructing composites that dissipate impact energy from supersonic impacts.

## Conflicts of interest

The authors declare no conflict of interest.

## Supplementary Material

RA-010-C9RA09685J-s001

RA-010-C9RA09685J-s002

RA-010-C9RA09685J-s003

RA-010-C9RA09685J-s004

RA-010-C9RA09685J-s005
