# Supplementary material for: High impact resistance in graphyne
Source: RSC Adv. 2020 Jan 9;10(3):1697–703. doi: 10.1039/c9ra09685j (PMC9048187; doi:10.1039/c9ra09685j)
Supplement: RA-010-C9RA09685J-s005 [file RA-010-C9RA09685J-s005.pdf]

Electronic Supplementary Material (ESI) for RSC Advances.
This journal is © The Royal Society of Chemistry 2019

Electronic Supplementary Information (ESI)

High impact resistance in graphyne

Yang Yang,[a, b] Qiang Cao,*[a,b] Yang Gao,[c] Shuting Lei,[a, b] Sheng Liu[a, b] and Qing Peng *[a,b,d]

Author affiliations

[a] The Institute of Technological Sciences, Wuhan University, Wuhan 430072, China

[b] Key Laboratory of Hydraulic Machinery Transient, Ministry of Education, Wuhan University

[c] Science and Technology on Special System Simulation Laboratory, Beijing Simulation Center, Beijing, 100854, PR China

[d] Nuclear Engineering and Radiological Sciences, University of Michigan, Ann Arbor, MI 48109, USA

* Corresponding authors: E-mail: caoqiang@whu.edu.cn, qpeng@umich.edu

1. The arrangement of marked points on the graphyne monolayer

Starting at 2 nm from the centre of graphyne monolayer, points are marked every 2 nm interval along X-direction, from X1 to X9. At 20 nm from the centre, X10 to X13, are marked every 3 nm interval along X direction respectively. Points in the Y direction are arranged the same way as points in the X direction.

Fig. S1 The arrangements of marked points on the graphyne monolayer. The length values in the figure are the distance measured from the origin. The points 1 - 9 in both directions are used to calculate the velocity of cone wave propagation, while 10 - 13 are for elastic wave propagation.

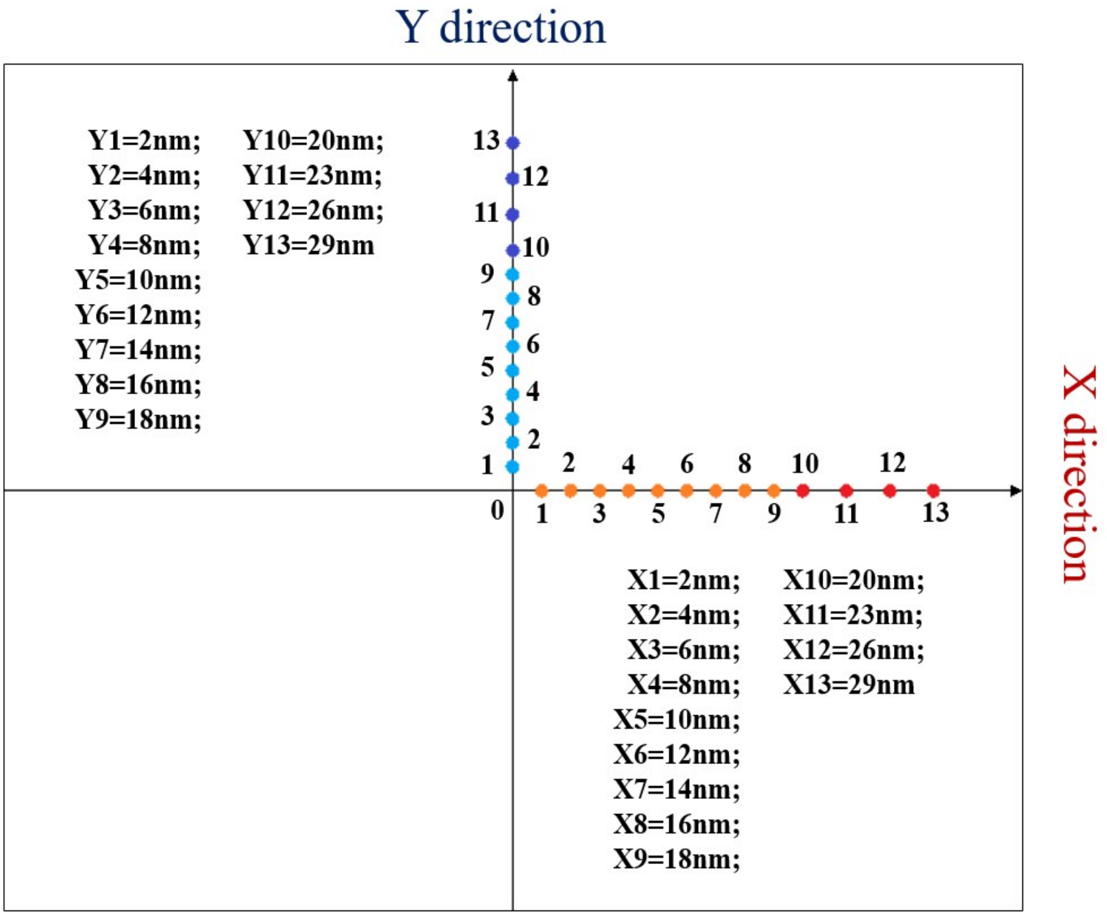

2. Figures of the velocities of elastic wave propagation

The velocities of elastic (sound) wave propagation from our simulation and membrane theory are drawn in the following figures for comparison.

Fig. S2 The velocities of elastic wave propagation. (a) α-graphyne; (b) β-graphyne; (c) δ-graphyne; (d) γ-graphyne. As the figures show, circle points represent the simulation values and the diamond points represent the theoretical values.

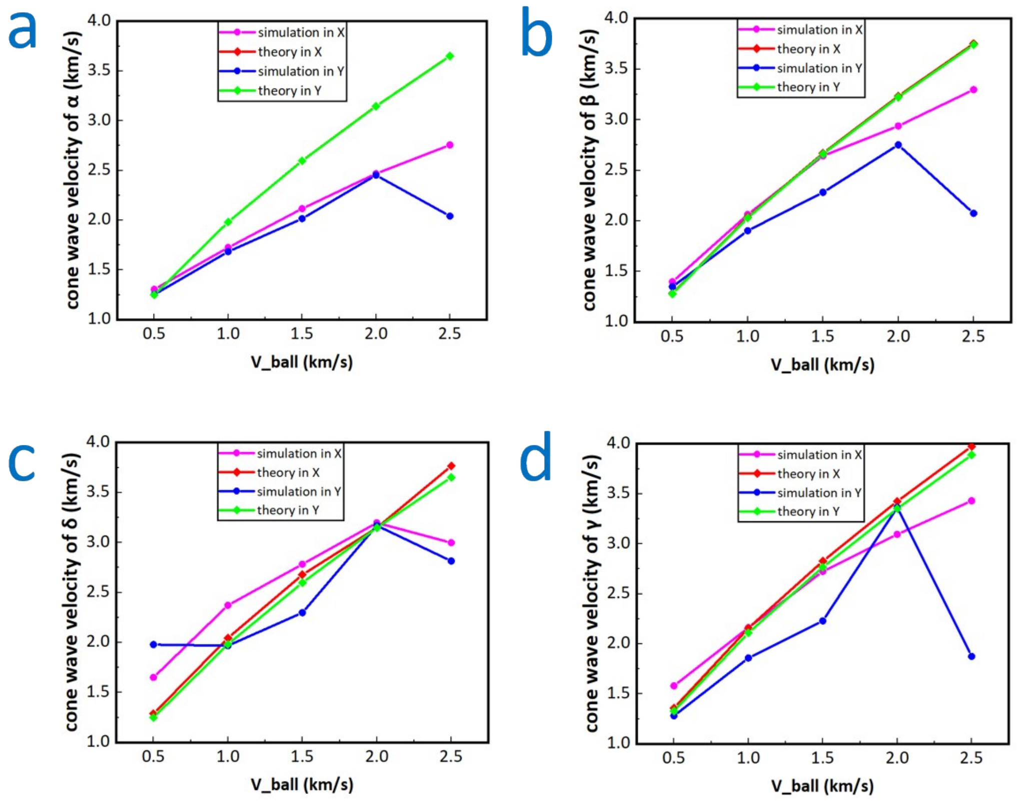

3. Detailed data of the velocities of elastic wave propagation

The detailed values of elastic wave propagation velocities are listed in the table, while the values of others are also listed for comparison.

Table S1. The velocities of elastic wave propagation. The values are in brackets are from Xia et al.[1] The values of graphene are from Haque et al.[2]

Graphyne Type	Simulation values in X direction	Theory values in X direction	Simulation values in Y direction	Theory values in Y direction
α-graphyne	16.281 (14.49)	15.564	12.422 (15.76)	15.548
β-graphyne	14.647 (15.61)	16.896	17.074 (16.34)	16.748
δ-graphyne	14.933 (17.65)	17.079	12.408 (14.71)	15.576
γ-graphyne	20.935 (17.90)	20.079	14.256 (17.90)	18.802
Graphene	18.40	21.22	18.01	21.19

4. Detailed data of the velocities of cone wave propagation

All the detailed velocities of cone wave propagation are listed here.

Table S2. The velocities of cone wave propagation

Vp (km/s)	α-graphyne		β-graphyne		δ-graphyne		γ-graphyne	
	simulation	theory	simulation	theory	simulation	theory	simulation	theory
0.5	c_{k1}=1.30<br>c_{k2}=1.25	c_{k1}=1.25<br>c_{k2}=1.25	c_{k1}=1.40<br>c_{k2}=1.35	c_{k1}=1.28<br>c_{k2}=1.28	c_{k1}=1.65<br>c_{k2}=1.98	c_{k1}=1.29<br>c_{k2}=1.25	c_{k1}=1.58<br>c_{k2}=1.28	c_{k1}=1.36<br>c_{k2}=1.33
1.0	c_{k1}=1.73<br>c_{k2}=1.68	c_{k1}=1.98<br>c_{k2}=1.98	c_{k1}=2.06<br>c_{k2}=1.90	c_{k1}=2.04<br>c_{k2}=2.03	c_{k1}=2.37<br>c_{k2}=1.97	c_{k1}=2.04<br>c_{k2}=1.98	c_{k1}=2.16<br>c_{k2}=1.86	c_{k1}=2.16<br>c_{k2}=2.11
1.5	c_{k1}=2.12<br>c_{k2}=2.02	c_{k1}=2.59<br>c_{k2}=2.60	c_{k1}=2.64<br>c_{k2}=2.28	c_{k1}=2.67<br>c_{k2}=2.66	c_{k1}=2.78<br>c_{k2}=2.30	c_{k1}=2.68<br>c_{k2}=2.60	c_{k1}=2.72<br>c_{k2}=2.23	c_{k1}=2.83<br>c_{k2}=2.77
2.0	c_{k1}=2.47<br>c_{k2}=2.45	c_{k1}=3.15<br>c_{k2}=3.14	c_{k1}=2.94<br>c_{k2}=2.75	c_{k1}=3.23<br>c_{k2}=3.22	c_{k1}=3.20<br>c_{k2}=3.17	c_{k1}=3.15<br>c_{k2}=3.15	c_{k1}=3.09<br>c_{k2}=3.36	c_{k1}=3.42<br>c_{k2}=3.35
2.5	c_{k1}=2.76<br>c_{k2}=2.04	c_{k1}=3.65<br>c_{k2}=3.65	c_{k1}=3.30<br>c_{k2}=2.07	c_{k1}=3.75<br>c_{k2}=3.74	c_{k1}=1.65<br>c_{k2}=1.43	c_{k1}=3.77<br>c_{k2}=3.65	c_{k1}=3.43<br>c_{k2}=1.87	c_{k1}=3.97<br>c_{k2}=3.89

1. K. Xia, H. Zhan, A. Ji, J. Shao, Y. Gu and Z. Li, *Beilstein Journal of Nanotechnology*, 2019, **10**, 1588-1595.
2. B. Z. G. Haque, S. C. Chowdhury and J. W. Gillespie Jr, *Carbon*, 2016, **102**, 126-140.
